# Nonsurgical Aesthetic Treatment of the Face and Neck in GLP-1 Receptor Agonist Weight Loss Patients: Experience-Based Considerations

**DOI:** 10.1093/asjof/ojag011

**Published:** 2026-01-21

**Authors:** Amir Moradi, Radina Denkova, Katherine Holcomb, Anthony Rossi, Nazanin Ashourian

## Abstract

Glucagon-like peptide-1 (GLP-1)-based therapies are increasingly used to support weight loss. However, there can be significant sequelae with regard to the aesthetics of the face and neck, including midface volume loss; temple, infraorbital, and submalar hollowing; increased skin laxity; accentuation of wrinkles and folds; and worsened skin quality. Individuals seeking nonsurgical correction of these issues are likely to become increasingly common, but treatments have not yet been widely studied in this population. The aim of this paper is to provide experience-based guidance on multimodal nonsurgical aesthetic treatment of the face and neck in patients undergoing weight loss with GLP-1-based therapies. Four clinicians with extensive experience of this patient group completed a written questionnaire and were individually interviewed. Their joint knowledge and expertise form the basis of this guidance. In addition, a PubMed literature search was conducted up to March 31, 2025. Eligibility for nonsurgical modalities does not typically differ greatly in this group compared with other aesthetic patients, but consideration should be given to the optimal timing of treatment within the weight loss journey. Multimodal approaches are usually needed based on volumizing with hyaluronic acid fillers; skin tightening with collagen-stimulating methods (such as energy-based devices); reduction of wrinkles and banding (particularly in the lower face and neck); and other skin quality treatments. Regular follow-up is crucial for tracking outcomes and evaluating evolving aesthetic needs. Holistic management should incorporate detailed patient education and ongoing nutritional, lifestyle, and psychological support. By following these principles, good aesthetic outcomes can be achieved.

**Level of Evidence**: 5 (Therapeutic) 
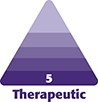

Concerns about body weight are a common problem throughout the world. Estimates from the World Health Organization suggest that 43% of all adults globally are overweight and 16% have obesity.^1^ In the US, the problem is even more pronounced—over 40% of adults are considered to have obesity, with associated costs totaling around $170 billion per year in medical expenditure alone.^2^

Lifestyle changes based on diet and exercise remain the cornerstone of sustainable weight loss. However, drug therapies are an increasingly important adjunct for many patients, particularly the glucagon-like peptide-1 (GLP-1) receptor agonists (as well as GLP-1 and glucose-dependent insulinotropic polypeptide co-agonists). These compounds were initially developed for the treatment of type 2 diabetes (T2D), but many have proven weight loss benefits in randomized controlled trials among individuals without T2D.^3-6^ As a result, several GLP-1-based therapies have been approved for use in weight management in the US, Europe, and elsewhere, including semaglutide, liraglutide, and tirzepatide.^7-12^ Their effects are primarily mediated through promoting satiety, suppressing gastric emptying, and reducing food intake.^13,14^

Although indicated only in patients with a body mass index of ≥30 kg/m^2^ (or ≥27 kg/m^2^ with at least one weight-related comorbidity), GLP-1-based weight loss therapies are commonly used off-label by individuals without obesity or overweight.^15^ In these instances, the primary motivation is likely to be aesthetic rather than health-related. Semaglutide in particular has been popularized through celebrity endorsements and social media influencers,^16^ and prescription volumes have increased rapidly in recent years.^17^ A recent survey of 368 aesthetic practitioners in the US found that a quarter were already using GLP-1-based weight loss therapies within their own practice.^15^

Importantly, the resulting weight loss is not specific to particular regions of the body and affects facial fat as well as that found elsewhere.^18^ Thus, beyond the obvious benefits of reducing excess weight, these treatments can have a significant detrimental impact on facial aesthetics. This may be psychologically damaging, particularly given that most aesthetically inclined individuals are at least as concerned about the appearance of their face as that of their body.^19^ The gauntness and skin laxity commonly resulting from GLP-1-based weight loss has been dubbed “Ozempic face”—after the brand name of one such product.^18,20^ The effects on appearance are often similar to the impact of aging.^21,22^ In a pilot study of aesthetic patients, blinded evaluation suggested that those with massive weight loss looked 5.1 years older than their actual age.^21^

In the coming years, individuals seeking correction of the negative consequences of GLP-1-based therapies are likely to form an increasingly large group worldwide.^20,23^ However, nonsurgical aesthetic treatments have not yet been extensively studied in this population. In the absence of such data, expert opinion is a particularly important driver of best practice.

The purpose of this paper is to provide experience-based guidance on multimodal nonsurgical aesthetic treatment of the face and neck in patients undergoing weight loss with GLP-1-based therapies.

## GUIDANCE DEVELOPMENT

In January 2025, the 4 authors who are aesthetic practitioners independently completed a written questionnaire on nonsurgical treatment of the face and neck in this patient group. A blank version of the survey is provided in the Appendix. It included 20 questions on the authors’ personal experience with such individuals, the aesthetic changes that can be addressed nonsurgically, patient selection, timing and methods for treatment, safety, and holistic patient management. Each author was then separately interviewed via video call to elaborate on their written responses. The present publication collates their joint knowledge and experience, based on diverse medical specialties (plastic surgery and dermatology) and practice settings (institutional and/or private practice in three different regions of the US and in Europe). Relevant case examples are provided. At the time of completing the questionnaire, the authors had personally managed around 250 nonsurgical facial aesthetic patients in total who were *in the process* of undergoing weight loss with a GLP-1-based therapy and at least 150 more who had *previously* undergone weight loss using these drugs.

## NARRATIVE LITERATURE REVIEW

In addition, to ensure that relevant previous publications were considered, a literature search of the PubMed database was conducted up to and including March 31, 2025. The search terms used were: (glucagon like peptide 1 OR GLP-1 OR exenatide OR Byetta OR Bydureon OR liraglutide OR Victoza OR Saxenda OR semaglutide OR Ozempic OR Rybelsus OR Wegovy OR albiglutide OR Tanzeum OR dulaglutide OR Trulicity OR lixisenatide OR Lyxumia OR Adlyxin OR tirzepatide OR Mounjaro OR Zepbound) AND weight AND (aesthetic OR esthetic OR cosmetic OR plastic surgery OR contour).

The search yielded 42 unique hits, which were then assessed for applicability based on consideration of aesthetic treatment in patients undergoing weight loss with GLP-1-based therapies. Other relevant publications were added through the personal knowledge of the author group. This gave a total of 22 published papers.^13,15,16,18,20,23-39^ However, the majority focused on surgical management, with few discussing nonsurgical approaches to the face and neck in these patients.^13,18,20,23,24,31,34,36^ Only one paper considered the full range of nonsurgical treatments in any detail,^13^ and none provided any data on aesthetic outcomes. A recent consensus report attempted to define parameters for understanding and managing the aesthetic needs of these patients.^36^ That paper was an important step forward but did not provide much guidance on key practical concerns around treatment planning and selection, safety, or holistic patient management.

## EXPERIENCE-BASED CONSIDERATIONS

### Aesthetic Needs

Substantial weight loss inevitably has physiological and structural consequences for the face and neck across multiple anatomical layers, including fat, skin, and muscle. Individuals undergoing GLP-1-based therapy may show large reductions in facial fat mass, including from superficial compartments.^13,40^ Further studies are required to assess the distribution of such losses but they appear not to be evenly spread. For example, volumetric analysis suggested that fat reductions were greater in the superficial buccal fat pads compared with the temporal fat pads.^40^

In addition, weight loss can have important effects on the skin. Although studies are limited, histological assessment found that collagen fibers were significantly thinner in the dermis of individuals with prior massive weight loss compared to those with no such history, and there was also apparent damage to the elastic fiber network.^41^ These issues may be particularly problematic among older patients, in whom collagen and elastin are usually already reduced.^24^

The weight loss effects of GLP-1-based therapies are mediated through increased satiety and lower food intake.^13,14^ Hence, there is a risk of undernourishment and micronutrient deficiency if patients fail to take in adequate amounts of key dietary components.^42,43^ Furthermore, depletion of fatty acids may affect skin barrier function, leading to dryness and dullness of appearance.^24^

GLP-1-based treatments may be associated not just with fat loss but also reduced lean mass, including possible muscular atrophy.^31,44^ Indeed, a network meta-analysis of randomized controlled trials of GLP-1 receptor agonists in patients with T2D noted significant decreases in fat-free mass.^45^ Studies in weight loss patients have also suggested that some lean mass may be lost.^44^

In addition to these “indirect” effects of GLP-1-based therapies caused by weight loss itself and by related nutritional deficits, it has been hypothesized that “direct” mechanisms might potentially be associated with structural changes to the face and neck. For example, these drugs may specifically promote the loss of dermal and subcutaneous white adipose tissue, alter the proliferation and differentiation of adipose-derived stem cells, and reduce the production of dermal estrogen and growth factors.^44^ Further studies are needed to assess such effects.

**Figure 1.**
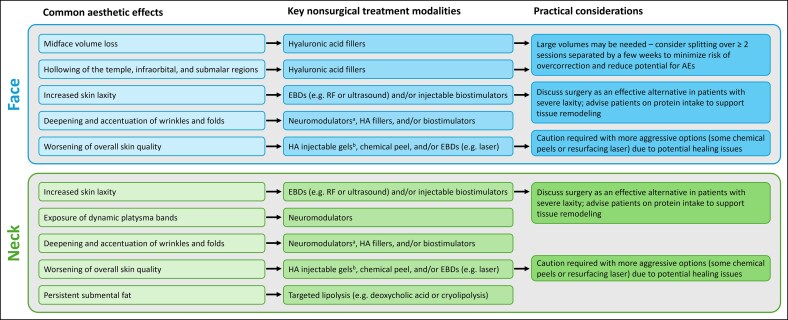
Common aesthetic effects of weight loss with GLP-1-based therapies and key nonsurgical treatment modalities. ^a^For dynamic lines only. ^b^With low G′ and low cohesivity. AE, adverse event; EBD, energy-based device; GLP-1, glucagon-like peptide-1; HA, hyaluronic acid; RF, radiofrequency.

This raft of changes is associated with various clinically significant but often treatable aesthetic effects on the face (Figure 1).^13,18,24,31^ These commonly include midface descent and loss of volume; hollowing of the temple, infraorbital, and submalar regions; increased skin laxity; deepening and accentuation of wrinkles and folds (eg, nasolabial folds, accordion or radial cheek lines, marionette lines); and worsening of skin quality (eg, dryness, crepiness, and dullness). Meanwhile, in the neck, analogous effects are frequently observed, including skin laxity, exposure of platysma bands, increased wrinkling, and worsening of skin quality.^13,31^

The severity of these changes in the face and neck can vary widely based on a number of factors. In our experience, these may include patient demographics (eg, age, sex, and ethnicity); baseline weight and the quantity and speed of loss; underlying anatomy, including face shape, bone structure, and muscle tone; baseline skin quality; genetic predisposition; lifestyle factors such as diet, exercise, smoking, and alcohol consumption; and other comorbidities. In particular, the trajectory of weight loss may be crucial, with more severe aesthetic changes typically observed in patients who lose greater quantities of weight at greater speed. In addition, younger patients often display less severe effects, whereas older individuals frequently have preexisting problems with midface volume loss and lax skin that are exacerbated by weight loss.

It is also important to note that the aesthetic effects of weight loss on the face and neck are not always negative. Reductions in fat mass may improve overall shape and contour in some individuals. Furthermore, we have observed that particular attributes of skin quality are sometimes improved. For example, general “puffiness” and edema may be reduced and there can also be improvements in inflammatory skin conditions like acne, atopic dermatitis, rosacea, and psoriasis—owing to the combined impact of weight loss, putative anti-inflammatory effects of GLP-1-based therapies,^14^ and other lifestyle improvements that may be implemented by some patients (eg, reduced alcohol consumption and smoking).

### Patient Selection and Treatment Planning

In our experience, the demographic profile of patients seeking nonsurgical treatment while undergoing weight loss with GLP-1-based therapies does not differ greatly from the wider aesthetic population—it is primarily females, mostly in midlife. However, this requires further study.

Furthermore, in the majority of cases, eligibility for nonsurgical treatment does not differ greatly in this group compared with non-GLP-1 patients. As with anyone presenting in clinic, it is essential to complete a thorough patient history and a comprehensive examination of the face and neck before proceeding, and to ensure that they have realistic expectations about what is possible—particularly if there are budgetary limitations or other constraints.

Practitioners should also bear in mind that GLP-1-based therapies are now widely available but not all patients will proactively divulge that they are using one. Furthermore, drug safety and effectiveness could be compromised if they are taking compounded medicines rather than the licensed versions.^46^ It is therefore important to ask specifically about these medicines when taking a medical history from all aesthetic patients.

**Figure 2.**
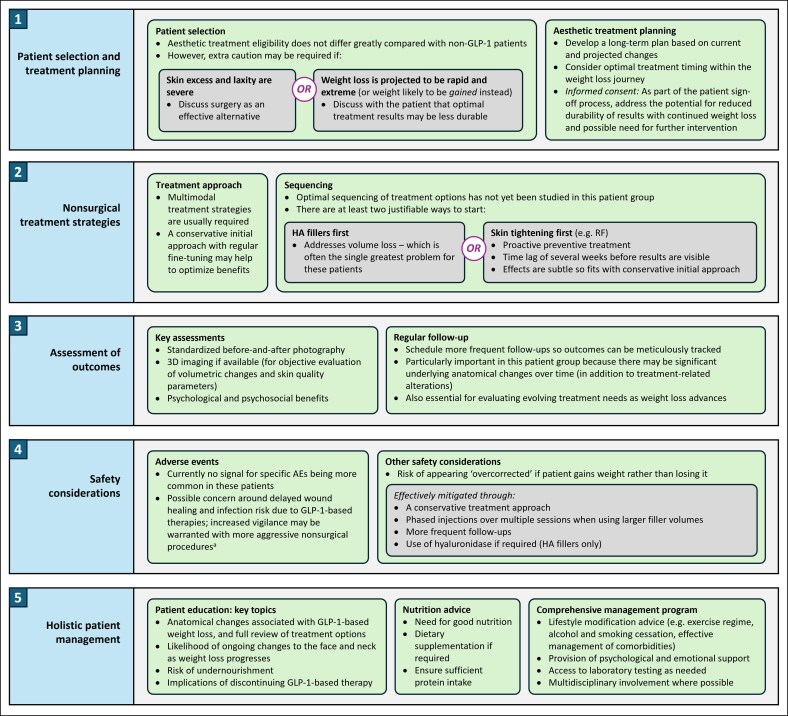
Key guidance on nonsurgical aesthetic treatment of the face and neck in patients undergoing weight loss with GLP-1-based therapies. ^a^Such as laser resurfacing or chemical peels. AE, adverse event; GLP-1, glucagon-like peptide-1; HA, hyaluronic acid; RF, radiofrequency.

We have noted 2 profiles with whom extra caution may be needed, although neither should be considered as an absolute contraindication for nonsurgical aesthetic treatment (Figure 2). The first relates to the degree of skin excess and laxity; where this is particularly severe, nonsurgical methods are unlikely to be sufficient to fully address patient needs, and surgery should be discussed as a potential alternative (with nonsurgical procedures as possible adjuncts). The second relates to the likely weight trajectory. It is reasonable to plan treatment based on steady losses but if a practitioner suspects that weight loss will be extreme, or alternatively that weight will actually be gained rather than lost, aesthetic outcomes may be compromised. Before proceeding with these patients, it is particularly important to discuss the potential for less durable results and the likely need for additional treatments.

The timing of aesthetic interventions within the weight loss journey is a key consideration and should be a shared decision between practitioner and patient, bearing in mind patient motivations. Some individuals are very focused on immediate improvements to mediate against the worst extremes of face and neck skeletonization; others may wish to wait until their weight stabilizes to avoid over- or under-correction and allow for more predictable results.

In patients wanting aesthetic treatment while weight loss is ongoing, we believe that informed consent is a key nonclinical consideration. Given the potential for reduced durability of results owing to continued weight loss, and hence the possible need for additional interventions, practitioners may wish to incorporate explicit understanding of this within their patient consent process.

Before starting treatment, it is important to educate patients fully and develop a long-term plan based on current and projected changes. Frequent follow-up visits should be built into this plan to facilitate enhanced monitoring of aesthetic and weight loss outcomes, and allow for adjustments or further correction as necessary. In many cases, a conservative initial approach followed by regular fine-tuning may help to optimize benefits.

### Nonsurgical Treatment Strategies

**Figure 3.**
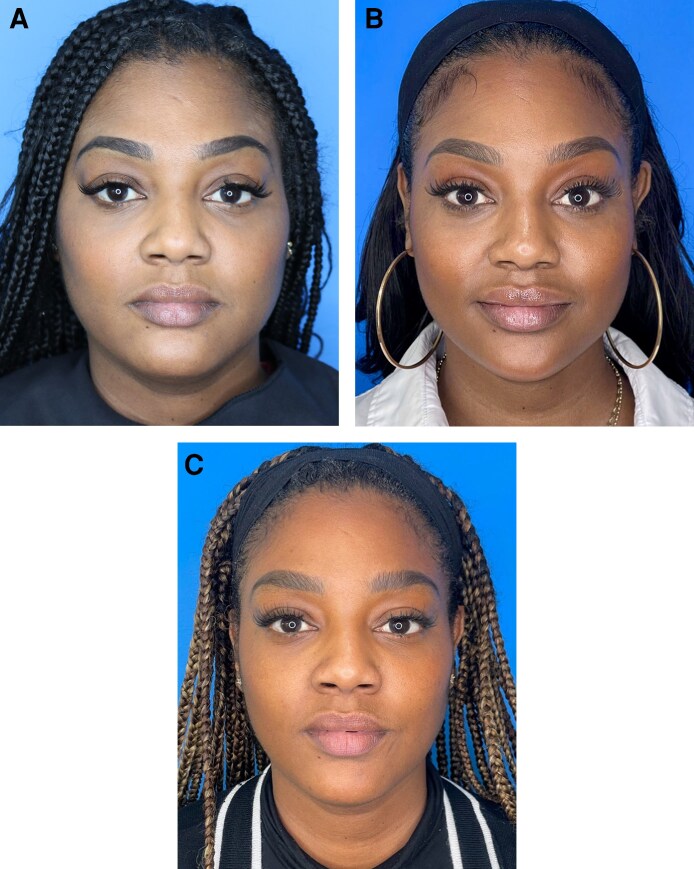
A 39-year-old female who received multimodal nonsurgical aesthetic treatment during and after weight loss with a GLP-1-based therapy. The patient lost a total of 40 lb (∼18 kg) over a period of 12 months. During weight loss, she was treated with neuromodulator in the neck, glabella, and crow's feet. Following weight loss, she was treated with HA filler in the cheeks (1 mL per side), jawline (1 mL per side), and chin (1 mL total); and neuromodulator in the chin, neck, glabella, and crow's feet lines. She is shown (A) prior to weight loss, (B) mid-weight loss (following neuromodulator treatment; 4 months after the baseline photograph; 18 lb lost to that point), and (C) after all weight loss and 5 months on from aesthetic treatment. Images courtesy of Dr Amir Moradi. GLP-1, glucagon-like peptide-1; HA, hyaluronic acid.

**Figure 4.**
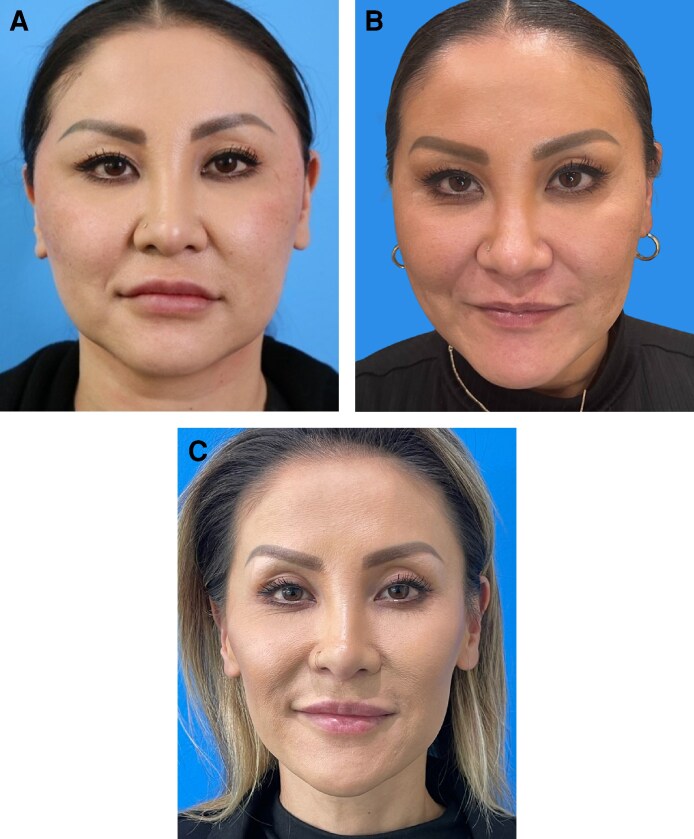
A 42-year-old female who received multimodal nonsurgical aesthetic treatment during and after weight loss with a GLP-1-based therapy. The patient lost a total of 40 lb (∼18 kg) over a period of 10 months. During weight loss, she was treated with neuromodulator in the neck. Following weight loss, she was treated with HA fillers in the lips (2 mL per side), jawline (1 mL per side), and chin (1 mL total), as well as for acne scars (1 mL per side); and with neuromodulator in the neck, masseter, crow's feet, and glabella. She is shown (A) prior to weight loss, (B) mid-weight loss (following neuromodulator treatment; 3 months after the baseline photograph; 15 lb lost to that point), and (C) after all weight loss and 4 months on from aesthetic treatment. Images courtesy of Dr Amir Moradi. GLP-1, glucagon-like peptide-1; HA, hyaluronic acid.

**Figure 5.**
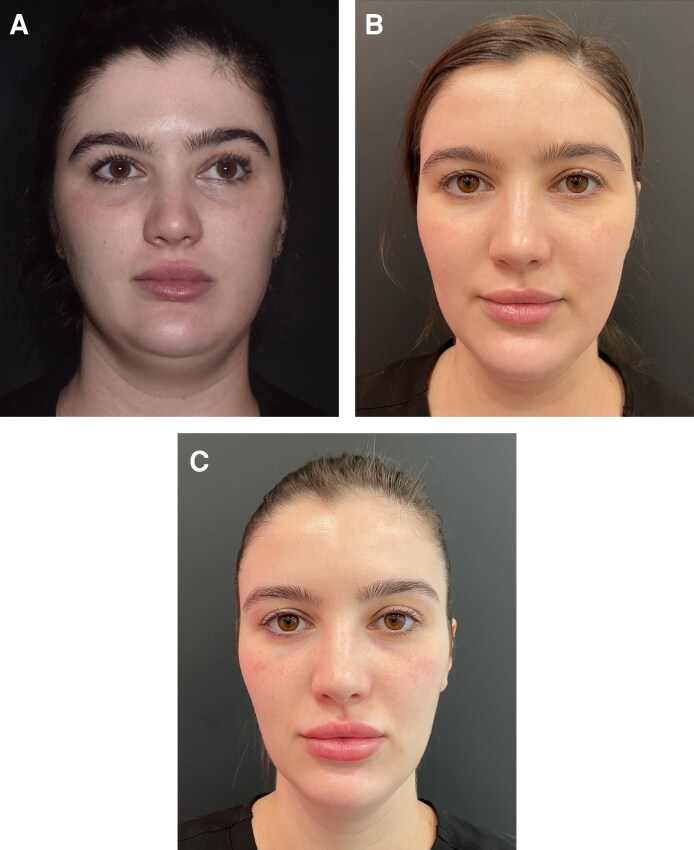
A 29-year-old female who received multimodal nonsurgical aesthetic treatment during and after weight loss with a GLP-1-based therapy. The patient lost a total of 32 lb (∼15 kg) over a period of 19 months. She is shown (A) prior to weight loss, (B) after 18 lb of weight loss (6 months after the baseline photograph), and (C) after an additional 14 lb of weight loss (16 months after the baseline photograph). Between (A) and (B), she was treated with neuromodulator in the upper face and masseter (for TMJ pain), injectable HA-based skin-quality therapy (3 mL), and biostimulator (PLLA) to the face (jawline, cheeks, lateral chin, temples; 18 mL total) and neck (4.5 mL). Between (B) and (C), she was treated with neuromodulator in the upper face and masseter (for TMJ pain); injectable HA-based skin-quality therapy (2.5 mL); biostimulator (PLLA) to the face (jawline, cheeks, lateral chin, temples; 13.5 mL total) and neck (4.5 mL); 532 nm laser for erythema and telangiectasias; radiofrequency treatment for submental fat reduction; and HA filler in the nasolabial folds (0.2 mL), marionette lines (0.2 mL), lateral chin (0.6 mL), and lips (1.0 mL). In part (C), the patient is at a stable weight and ≥ 2 months on from all of the aesthetic treatments listed. Images courtesy of Dr Katherine Holcomb. GLP-1, glucagon-like peptide-1; HA, hyaluronic acid; PLLA, poly-L-lactic acid; TMJ, temporomandibular joint.

**Figure 6.**
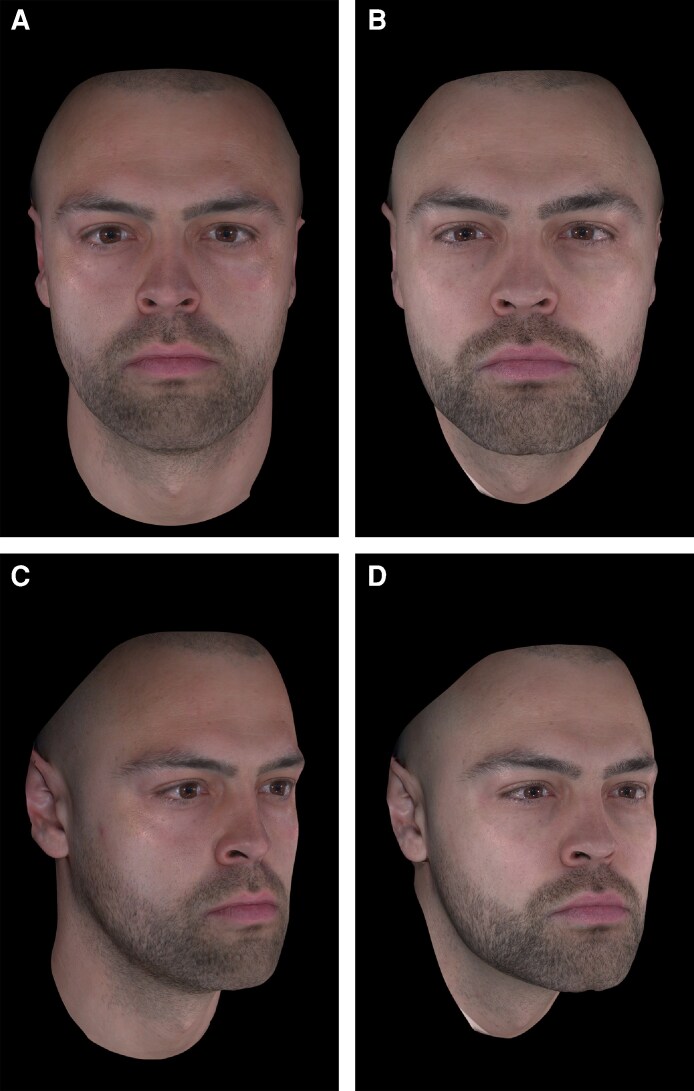
A 32-year-old male who received multimodal nonsurgical aesthetic treatment following weight loss with a GLP-1-based therapy. The patient lost a total of 19 kg (∼42 lb) over a period of 9 months. He was then treated with HA fillers in the temples (1 mL total), cheeks (4 mL total), chin (2 mL total), and jawline (4 mL total); and a hybrid product (containing both CaHA and HA) in the cheeks (1 mL total) and jawline (1.5 mL total). He is shown (A, C) following weight loss but before aesthetic treatment and then (B, D) 6 weeks after aesthetic treatment. Images courtesy of Dr Radina Denkova. CaHA, calcium hydroxyapatite; GLP-1, glucagon-like peptide-1; HA, hyaluronic acid.

In our experience, the full range of nonsurgical modalities should be considered in weight loss patients. However, the final treatment plan must be personalized according to the specific deficits that require addressing or are likely to need rectification in future. Multimodal approaches are usually favored (Figure 1). Examples demonstrating the impact of aesthetic treatment in individual patients are provided in Figures 3-6. All provided written informed consent for treatment and for their images to be used in this publication.

In many cases, we have found that the key priority is volumizing and contouring of the midface, where volume loss is often particularly pronounced. Treatment of this area can also have indirect benefits across the whole face.^47^ For example, cheek augmentation with a hyaluronic acid (HA) filler in individuals with midface volume deficit was found to improve patient satisfaction not only with their cheeks but also other (untreated) areas, such as the tear troughs and nasolabial folds.^48^ Additional priority treatment areas where volume loss is most prominent may include the temple, jawline, and periorbital areas. HA fillers with medium-to-high G′ and cohesivity will typically be required to provide sufficient lift and structure, although products with more subtle lift capacity may sometimes be preferable (eg, in the periorbital area). Detailed guidance has been published elsewhere describing the rheological properties of different HA fillers and the relationship with clinical performance.^49^ The amount of filler required will depend on the degree of volume loss. For some patients with significant volume loss, full correction may be best achieved by performing the treatment over 2 or more sessions separated by at least a few weeks in order to minimize the risk of overcorrection and reduce the potential for adverse events.

A second priority area for many patients will be skin tightening to address laxity, particularly in the cheek and neck. As already noted, nonsurgical approaches are unlikely to be sufficient in those with severe problems. However, for individuals with mild-to-moderate laxity, energy-based device (EBD) treatments are often useful, such as radiofrequency (with or without microneedling) or ultrasound. These technologies work primarily by stimulating collagen contraction in the deep dermis, leading to the development of new collagen and the shrinking of high-elasticity tissues.^50^ Injectable “biostimulator” products can also be employed for this purpose, for example those based on calcium hydroxyapatite (CaHA), poly-L-lactic acid, or polycaprolactone.^51,52^ Hypothetically, skin tightening with collagen-stimulating methods might have a reduced impact if patients are undernourished and absorbing insufficient protein to support tissue remodeling, although this has not yet been analyzed in clinical studies. When using injectable biostimulators, it may therefore be preferable to use products that combine collagen stimulation with direct volumization. For example, a hybrid product (HA-CaHA) containing both CaHA for collagen stimulation and HA for immediate volume and lift has been shown to be safe and effective in large real-world analyses of aesthetic patients.^53,54^

In our experience, a third key priority for many GLP-1-based weight loss patients is the reduction of wrinkles and banding, particularly in the lower face and neck. For example, exposure of platysma bands is frequently a concern. Neuromodulators are often the key treatment modality. OnabotulinumtoxinA is the first such product to be approved by the US Food and Drug Administration for the treatment of moderate-to-severe platysma bands, following positive results from two Phase 3 randomized controlled trials.^55,56^ Neuromodulator-based techniques can also be used to treat other key areas, such as the chin and marionette lines.^57,58^ However, these have not been studied in prospective clinical trials and are currently off-label.

In addition, HA products can improve the overall appearance of wrinkles and folds in the lower face and neck. Typically, these should have medium-to-low G′ and low cohesivity, allowing for easy molding and spread following superficial injection.^49^ For example, there is evidence of effects on horizontal neck lines using VYC-12L (SKINVIVE™ by JUVÉDERM®, Allergan Aesthetics, an AbbVie company, Pringy, Annecy, France),^59,60^ and this is now being assessed in larger studies.^61^

HA products may also have significant value in treating another important issue in some patients—reductions in skin quality, particularly relating to dryness and dullness. VYC-12L has been shown to improve skin hydration and smoothness in the face and neck,^59,62^ and is the only HA injectable product with hydration benefits included in its label.^63^ Other skin quality treatments include more aggressive options like some chemical peels or laser resurfacing, although these methods rely on efficient healing, which may be challenging if patients are undernourished. In practice, issues like skin dryness and roughness are often a less pressing concern than volume loss, laxity, and wrinkle accentuation. However, they may become more important as weight stabilizes and patients’ facial appearance improves with treatment.

Somewhat paradoxically, a final nonsurgical treatment that we believe should be considered in some patients is targeted lipolysis, for example using deoxycholic acid or cryolipolysis.^64,65^ These methods can be used to address stubborn pockets of fat that sometimes persist, particularly in the submental region, even after extensive fat loss in other parts of the face and neck.

The optimal sequencing of all these treatment options has not yet been studied in GLP-1-based weight loss patients. Within the current author group, some favor starting with HA fillers to address volume loss, which is often the single greatest problem for these patients; other authors prefer to begin with proactive skin tightening (eg, radiofrequency) because there is a time lag of several weeks before results are visible and because the effects are subtle so it fits well with a conservative initial approach. Different modalities can sometimes be combined within the same session or may be used separately, as per the specific needs of the individual patient.

### Assessment of Outcomes

As with any patient receiving nonsurgical aesthetic treatment, it is important to track outcomes over time (Figure 2). This can be achieved using standardized before-and-after photography. Furthermore, various 3D imaging systems are available that provide objective analyses of volumetric changes and skin quality parameters, which are particularly relevant assessments in this group. Alongside aesthetic and safety outcomes, practitioners should consider recording patients’ weight at each visit and should assess psychological and psychosocial effects (informally or based on relevant questionnaires) in order to build a holistic understanding of progress and patient wellness.

In addition, it is important to schedule regular follow-ups so that all outcomes can be meticulously tracked. Frequent and accurate monitoring is especially important with these patients given that there may be significant underlying anatomical changes over time (in addition to treatment-related alterations). This will also be essential for evaluating their evolving treatment needs as weight loss advances.

### Safety Considerations

The safety of nonsurgical aesthetic treatment has not yet been formally assessed in clinical studies of patients using GLP-1-based therapies. However, in our practical experience, we have observed no specific adverse events that appear to be more common in this population as compared with other aesthetic patients. Hypothetically, it is possible that recovery times could be longer if individuals are undernourished and have significant dietary deficiencies. In addition, the putative effects of GLP-1-based therapies on platelet aggregation^66^ might increase bruising risk. However, we have not observed this ourselves.

In surgical settings, there have been reports of delayed wound healing and increased rates of surgical site infections in patients using GLP-1-based therapies prior to the procedure.^29,39^ These issues are unlikely to be highly relevant to minimally invasive, nonsurgical treatments, but increased vigilance may be warranted with more aggressive procedures like laser resurfacing or chemical peels.

A pertinent consideration with filler products is the risk of patients appearing “overcorrected” if they gain weight rather than losing it. Where there is concern, we have found that these risks can be effectively mitigated through a conservative treatment approach, phased injections over multiple sessions when using larger volumes, and more frequent follow-up. In addition, hyaluronidase can be used to degrade HA fillers in those individuals who do gain weight, if they then appear overcorrected.

### Holistic Patient Management

In our experience, education and support are particularly important in this group. In addition to the usual education that practitioners should undertake with all aesthetic patients, there are some additional needs among individuals taking GLP-1-based therapies. Key messages should be reinforced at each visit. Important topics for discussion include the specific anatomical changes associated with GLP-1-based weight loss and full review of appropriate treatment options; the likelihood of continuous, ongoing changes to the face and neck as weight loss progresses, which may negatively affect the duration of outcomes; and the need for good nutrition and possibly also for dietary supplementation to support optimal results. Given that some of the weight loss will likely come from lean body mass, patients might need to build new eating habits to ensure sufficient protein intake, equivalent to at least the recommended daily allowance of 0.8 g/kg.^67^

Furthermore, it is often worthwhile to discuss additional lifestyle modifications that can support both weight loss and aesthetic treatment outcomes, particularly among individuals who are using GLP-1-based treatments without any other medical supervision. Such modifications include healthy exercise regimes, alcohol and smoking cessation, and effective management of comorbidities. Appropriate fitness education may also help to reduce associated bone and muscle changes.

In addition, it may be valuable to discuss the potential implications of discontinuing GLP-1-based therapies. Trial extension data showed that participants regained around two thirds of previously lost weight within 1 year following withdrawal of the medication.^68^ In our experience, many patients are not aware of this risk, nor of the possible implications for aesthetic treatment outcomes should significant weight be gained.

We have found that psychological and emotional support is also important. These patients will likely have entered into GLP-1-based weight loss with an idea of looking healthier and younger; from a psychological readiness standpoint, many will be ill-prepared for facial volume loss, laxity, decreased skin quality, and an aged appearance. This disconnection between expectation and reality may have significant detrimental effects on self-esteem, confidence, and quality of life.

Relevant laboratory tests should also be considered, and many aesthetic practitioners now do this as part of standard practice, both on initiation and during the course of GLP-1-based weight loss.^15^ Commonly used tests include complete metabolic panels, complete blood counts, thyroid and lipid panels, hemoglobin A1c, vitamin D, and prealbumin.^15^

Overall, these patients should ideally have comprehensive lifestyle plans, as well as multispecialty physical and psychological management. Although aesthetic practitioners cannot typically be personally responsible for delivering all of the above, they should consider how best to collaborate with other relevant specialties to facilitate holistic care. Furthermore, regular touchpoints and open lines of patient communication may help to establish positive, sustainable habits and support long-term outcomes.

### Limitations and Future Directions

There are limitations to the experience-based guidance provided here for nonsurgical aesthetic treatment in patients undergoing GLP-1-based weight loss. For example, there is currently no consensus definition around the quantity or speed of such weight loss that delineates the clinical need for population-specific aesthetic considerations. This is an area for further study but, regardless, we believe our guidance is relevant to the great majority of such patients. In addition, it is important to note that these considerations relate to *nonsurgical* aesthetic treatment and should not be extrapolated to plastic surgery patients (or to those undergoing more invasive nonsurgical procedures like fat transfer). Specific guidance for plastic surgery in GLP-1-based weight loss patients is available elsewhere.^24,30,31,34,37,38^ Furthermore, the scope of this publication does not include other modes of medically assisted weight loss, such as bariatric surgery. We also acknowledge that the narrative literature review performed to collate previous publications could have missed some relevant papers, although this is a limitation of any such search.

The most important limitation with regard to nonsurgical treatment of GLP-1-based weight loss patients is the current lack of clinical data specific to these individuals. Such an absence is not surprising given that this is still a relatively new aesthetic population—but inevitably means that much of the guidance is derived from expert opinion rather than from study outcomes. This is of course a limitation of the entire field and is not exclusive to this publication.

There is now a pressing need for data collection, and many important scientific questions that still need to be addressed. Table 1 provides detailed suggestions on key future directions for clinical study in this area. In particular, prospective trials and large “real world” studies should be prioritized to confirm the utility of currently available treatments in GLP-1-based weight loss patients.

**Table 1.** Nonsurgical Aesthetic Treatment of the Face and Neck in GLP-1-based Weight Loss Patients: Future Directions

*Aesthetic treatments* High-quality clinical studies of safety, effectiveness, and psychosocial outcomes with nonsurgical aesthetic treatments in this population Sequencing and combination studies to evaluate the optimal order of nonsurgical treatments Assessment of the optimal timing of treatment within the weight loss journey (ie, intervening early vs waiting until weight has stabilized) Development of novel nonsurgical skin-laxity treatments that can improve on currently available options
*Supportive measures* Research on preventative methods for minimizing aesthetic changes during GLP-1-based weight loss Analysis of the impact of nutritional counseling and other lifestyle advice on treatment outcomes
*Patient profiles* Large-scale auditing of patient demographics within the GLP-1-based weight loss population seeking aesthetic treatment (sex, age, ethnicity, etc)
*Underlying changes* Greater understanding of the specific physiological and anatomical changes occurring in patients treated with GLP-1-based therapies (including effects on the skin, muscle, and fat) Assessment of the long-term impacts of GLP-1-based therapies

GLP-1, glucagon-like peptide-1.

## CONCLUSIONS

GLP-1-based therapies are increasingly being used to support weight loss but can have significant and often negative aesthetic consequences on the face and neck. Nonsurgical modalities such as HA fillers, neuromodulators, and collagen stimulators can improve such problems. The present publication provides experience-based guidance on managing these individuals (Figure 2). Key considerations include optimizing the timing of treatment within the weight loss journey, the deployment of multimodal approaches, frequent follow up, and holistic management incorporating nutritional, lifestyle, and psychological support. In addition, patient education and expectation control are essential. By following these principles, good aesthetic outcomes can be achieved.

## Supplemental Material

This article contains supplemental material located online at https://doi.org/10.1093/asjof/ojag011.

## Supplementary Material

ojag011_Supplementary_Data
