# Supplementary material for: Nonsurgical Aesthetic Treatment of the Face and Neck in GLP-1 Receptor Agonist Weight Loss Patients: Experience-Based Considerations
Source: Aesthet Surg J Open Forum. 2026 Jan 21;8:ojag011. doi: 10.1093/asjof/ojag011 (PMC12937588; doi:10.1093/asjof/ojag011)
Supplement: ojag011_Supplementary_Data [file ojag011_supplementary_data.pdf]

QUESTIONNAIRE: Nonsurgical aesthetic treatment of the face and neck in patients undergoing rapid weight loss with GLP-1-based therapies

PERSONAL EXPERIENCE

Question 1. Approximately how many nonsurgical aesthetic patients have you treated in the face / neck who were in *the process of* undergoing weight loss with a GLP-1-based therapy (e.g. semaglutide, liraglutide, tirzepatide)?

Question 2. Approximately how many nonsurgical aesthetic patients have you treated in the face / neck who had *previously* undergone weight loss with a GLP-1-based therapy (and had already reached their desired weight)?

Question 3. Are these individuals typically patients you were already treating (who subsequently started on GLP-1-based weight loss) or are they usually new to you? If they are usually new to you, are they normally treatment-naïve or have they received previous aesthetic treatment elsewhere?

Question 4. Have you noticed any demographic patterns within this patient group (age, sex, etc)?

AESTHETIC CHANGES THAT CAN BE ADDRESSED WITH NONSURGICAL TREATMENTS

Question 5. What aesthetic changes to the *face* do these patients typically show that can be improved using nonsurgical methods?

Question 6. What aesthetic changes to the *neck* do these patients typically show that can be improved using nonsurgical methods?

© Allergan Aesthetics COMPANY CONFIDENTIAL AND PROPRIETARY INFORMATION

Question 7. Does the severity of such changes depend on any specific factors (e.g. amount of weight loss, patient age, etc)?

PATIENT SELECTION AND TIMING OF TREATMENT

Question 8. How do you select patients undergoing GLP-1-based weight loss who are appropriate for nonsurgical aesthetic treatment? Would any individuals be ineligible?

Question 9. Do you typically start aesthetic treatment during rapid weight loss itself, or do you wait until the patient's weight has stabilized? What is your rationale for this choice of timing?

TREATMENT METHODS

Question 10. Which injectable treatments do you typically use to manage these individuals (product types, treatment areas, volumes, techniques, etc)?

Question 11. Apart from injectables, which other nonsurgical methods do you use to manage these individuals?

Question 12. In what order do you normally provide nonsurgical treatments?

© Allergan Aesthetics COMPANY CONFIDENTIAL AND PROPRIETARY INFORMATION

Question 13. Are there any differences in your treatment choices (modalities, techniques, etc) for individuals undergoing GLP-1-based weight loss compared with your other aesthetic patients?

Question 14. Do you have any practical tips or clinical pearls to share with other practitioners for treating these patients?

Question 15. Are there any differences in how you assess treatment outcomes with individuals undergoing GLP-1-based weight loss compared to your other patients? If so, how?

SAFETY

Question 16. Are there specific safety concerns or adverse events that practitioners should be particularly watchful for when treating these patients?

Question 17. If you answered 'yes' to the previous question, how can such risks be minimized?

PATIENT MANAGEMENT

Question 18. Are there any differences in the education / information needs of this type of patient?

Question 19. Are there any differences in post-treatment management and follow-up with this type of patient?

© Allergan Aesthetics COMPANY CONFIDENTIAL AND PROPRIETARY INFORMATION

Question 20. Are there any nonclinical issues that practitioners should be aware of (e.g. different informed consent forms, given that the face may change significantly during weight loss?)

© Allergan Aesthetics COMPANY CONFIDENTIAL AND PROPRIETARY INFORMATION
